# Dynamics of a Complex Multilayer Polymer Network: Mechanical Relaxation and Energy Transfer

**DOI:** 10.3390/polym10020164

**Published:** 2018-02-08

**Authors:** Aurel Jurjiu, Flaviu Turcu, Mircea Galiceanu

**Affiliations:** 1 Faculty of Physics, Babes-Bolyai University, Street Mihail Kogalniceanu 1, 400084 Cluj-Napoca, Romania; flavius.turcu@phys.ubbcluj.ro; 2 Department of Physics, Federal University of Amazonas, 69077-000 Manaus, Brazil; mircea@ufam.edu.br

**Keywords:** multilayer network, dynamics, rheological quantities, fluorescence depolarization, generalized Gaussian structures

## Abstract

In this paper, we focus on the mechanical relaxation of a multilayer polymer network built by connecting identical layers that have, as underlying topologies, the dual Sierpinski gasket and the regular dendrimer. Additionally, we analyze the dynamics of dipolar energy transfer over a system of chromophores arranged in the form of a multilayer network. Both dynamical processes are studied in the framework of the generalized Gaussian structure (GSS) model. We develop a method whereby the whole eigenvalue spectrum of the connectivity matrix of the multilayer network can be determined iteratively, thereby rendering possible the analysis of the dynamics of networks consisting of a large number of layers. This fact allows us to study in detail the crossover from layer-like behavior to chain-like behavior. Remarkably, we highlight the existence of two bulk-like behaviors. The theoretical findings with respect to the decomposition of the intermediate domain of the relaxation quantities, as well as the chain-like behavior, are well supported by experimental results.

## 1. Introduction

One of the basic challenges in polymer physics is to unveil how the dynamical features of a polymeric material are related to its geometry. Originating from the seminal works of Rouse [1] and Zimm [2], who focused on the investigation of dilute solutions of linear polymers, the issue of relating the structure of a polymer to its dynamics is becoming of increasing importance because of the continuous advances in polymer synthesis. The fast development of modern technologies demands very versatile polymeric materials with the continuously changing requirements of the industry. Using modern strategies, such as controlled radical polymerizations [3,4,5], supramolecular polymerizations [6] or stepwise synthesis [7], new macromolecules or supramolecules with very complex architectures and tunable properties have been synthesized.

Among the polymers with precisely controlled molecular structures, of broad interest are the dendrimers [8,9,10,11,12,13,14,15,16,17,18,19,20]. These are a class of synthetic polymers that have monodispersed molecular weights and a well-defined highly branched structure, resembling a perfect Cayley tree. Their unique topological characteristics have been exploited in a wide range of applications, such as in biosensors [21], for catalysis [22], in medicine for drug delivery and gene therapy [23,24], and as intravascular contrast-enhancing agents for magnetic resonance imaging [25].

Far from being only pure abstract mathematical concepts, the fractals have proved to be important models for describing the structure and the dynamics of real systems. Examples are provided from different fields of science. Hierarchically organized surfaces are found in the endoplasmic reticulum [26], in mitochondria [27], and in other cell organelles [28]. Fractal properties have been reported for the formation of protein fibers [29] and for the organization of DNA into hierarchical structures [30]. Fractal models are widely used for describing disordered systems [31], growth phenomena [32], and chemical reactions controlled by diffusion [33]. Fractal constructs have led to the development of materials with demonstrated potentials as molecular batteries, switches, and optical display devices [34,35,36].

Important progress has been made during the past two decades regarding the self-assembly of synthetic macromolecules. Polymer structures with the shape of regular fractals have been synthesized. Newkome et al. [37] reported the formation of hexagonal structures that organize into larger hexagonal structures, resulting in the “hexagonal Sierpinski gasket”. Very recently, Shang et al. [38] reported the synthesis of a whole series of molecularly assembled and defect-free Sierpinski triangles.

In this paper, we extend the previous works [39,40,41,42,43,44,45,46,47,48] devoted to the study of the dynamics of polymers with complex architectures by considering another class of polymer systems, the “multilayer networks” or “networks of the networks” [49,50,51,52]. The multilayer structure on which we focus is built by connecting to each other identical layer. In turn, each layer represents a multihierarchical structure that was built by replicating the dual Sierpinski gasket in the shape of a regular dendrimer. The multilayer network dynamics is studied through the investigation of two different dynamical processes, the mechanical relaxation and the fluorescence depolarization under dipolar quasiresonant energy transfer. For describing the mechanical relaxation process, we analyze the dynamical behaviors of the storage and loss moduli. The dynamics of the energy transfer over a system of chromophores arranged in the form of our multilayer network is described by the average probability that a chromophore excited at time $t_{0}$ is (still or again) excited at time *t*.

We depict the multilayer polymer network through a Gaussian bead–spring model; this is the generalization of the Rouse and Zimm models, developed for polymer chains, to incorporate polymer systems with more complex geometries, and it is referred to as a generalized Gaussian structure (GGS) [15,53,54,55,56,57,58,59]. The GGS model does not account for excluded volume constraints or for entanglement effects. The excluded volume effects are often screened in rather dense media, such as dry polymer networks and polymer melts. In turn, the entanglement effects are negligible for polymer networks with high densities of cross-links, meaning that the network strands between the cross-link points are rather short. The advantage of using the GGS model is that it allows one to explore very efficiently the dynamical properties of arbitrarily connected polymers by making use of the eigenvalues and eigenvectors of the connectivity matrix. Remarkably, the connectivity matrix is also fundamental in describing the dynamics of energy transfer over a system of chromophores. A reasonable assumption we make is that the energy is resonantly exchanged only between nearest neighbors. This is based on the fact that for dipolar interactions, the Forster transfer rate obeys $w\left(r\right)=C/r^{6}$, where *r* is the relative distance between the chromophores involved, resulting clearly in that the nearest-neighbor transfer is by far dominant.

In the framework of the GGS model, the topological details of the investigated polymer system are unveiled only in the intermediate time/frequency domain of the dynamical quantities [39,40,41,42,43,44]. The intermediate domain is bounded by crossover regions. Because of the occasionally large cross-over regions, it is imperative to be able to compute the dynamical behavior for very large structures. Clearly, this leads to a very large connectivity matrix whose exact numerical diagonalization is practically impossible to perform. Remarkably, for our multilayer polymer network, we have developed an analytical method whereby the eigenvalues of its connectivity matrix are determined iteratively. This allows us to analyze, with the Rouse-type approach, the dynamics of multilayer networks consisting of a huge number of monomers (up to several billions). The connectivity matrix, representing the discrete version of the Laplacian operator, is extensively used in different areas of science, for instance, in graph theory applied to biological systems [60], reaction–diffusion systems [61], the dielectric relaxation functions [42], and the NMR relaxation functions [62,63]. From here, the importance of determining its eigenvalue spectrum results clearly.

In a very recent work [64], we have studied the mechanical relaxation dynamics of a multihierarchical structure built by replicating the dual Sierpinski gasket fractal in the shape of a regular dendrimer. In the context of our multilayer network, this represents one layer. In the Rouse-type approach, the conclusion drawn was that the multihierarchical structure still keeps the original behaviors of its constituent components. The intermediate time (or frequency) domain of the studied dynamical quantities separates into two distinct regions, each region displaying the original behavior of a constituent component of the multihierarchical structure. The challenge then is to show how the geometry of the multilayer structure affects the dynamical behavior of the constituent components.

As a result of the connection between layers, each monomer of a layer obtains an additional two connections, except those from the upper and the lower layer that obtain only one additional connection, which make them stiffer and may alter the dynamical properties. In this respect, important aspects have to be clarified. For instance, will we still see a purely dendrimer- or Sierpinski-like behavior on a certain time/frequency range, or will the individual behavior of the constituent components evolve to mixture-like behavior with the adding of more multihierarchical layers? When the number of layers in the structure is much larger than the number of monomers in a single layer, we expect the whole structure to manifest a chain-like behavior. Practically, each layer plays the role of a macromonomer in a linear chain. Therefore, the transition from a multihierarchical structure (one layer) to a linear chain will offer us the overview of the whole dynamical process.

The choice of the dendrimer and of the dual Sierpinski gasket as constituents of the multihierarchical layer is based on the fact, as exemplified in the first part of the introduction, that both are already obtained experimentally. Furthermore, multilayer networks similar to that proposed by us have been experimentally synthesized. Of these, we recall the cylindrical supramolecular dendrimers [65], the multilayered assemblies that are constructed through the layer-by-layer (LbL) deposition of dendrimers [66,67], the gel-like supramolecular networks (based on Pillararene [68] and based on cucurbit[7]uril-adamantane cross-linked supramolecular hydrogels [69]), the cylindrical micelles [70], and the crystalline polymers [71,72,73].

## 2. Generalized Gaussian Structures

The impact of a monomer’s connectivity on the physical properties of polymers can be rigorously calculated in the framework of the GGS model [15,53,54,55,56,57,58,59]. By assuming harmonic entropic forces between the flexible repeat units, many static and dynamic properties can be calculated from a matrix structure, describing the connectivity of the polymer. Given that the procedure of the GGS was explained in detail in [15,53,54,55,56,57,58,59], here we mainly summarize the basic concept and the main formulas concerning the relaxation dynamics patterns. A GGS consists of *N* identical beads subject to friction and connected to each other by entropic springs. These elastic (entropic) springs obey Gaussian statistics. In this model, the solvent or the surrounding medium is replaced by a continuum, which is felt by all the beads through the viscous friction and the stochastic (or random) forces. Here, we consider the simple case in which any bead of the GGS undergoes the same friction (friction constant ζ) with respect to the surrounding viscous medium. Each arrangement of monomers on the polymer system is given by the set of position vectors $\left\{R_{i}\right\}$, where $R_{i}\left(t\right)=\left(X_{i}\left(t\right),Y_{i}\left(t\right),Z_{i}\left(t\right)\right)$ represents the position vector of the *i*th monomer (bead) at time *t*, i=1,…,N. The potential energy of the GGS contains only the elastic contributions of nearest-neighbor monomers. Incorporating, also, the interactions with external forces $\left\{F_{n}\right\}$, the potential energy has the following expression:

(1) $$U\left(\left\{R_{i}\right\}\right)=\frac{K}{2}\sum_{\beta ,m,n}R_{\beta m}A_{nm}R_{\beta n}-\sum_{\beta ,n}F_{\beta n}R_{\beta n}$$

where, in the first sum, all bonds are considered to be equal with a length *l*, the mean distance between neighboring beads; *K* is the spring constant; β runs over the Cartesian coordinates *x*, *y*, and *z*; and the whole GGS configuration is accounted through the connectivity matrix $A=(A_{ij})$, a discrete version of the Laplacian operator. The connectivity matrix A is a real symmetric matrix, and it indicates which monomers are or are not directly bounded to each other. Its construction is very simple: the diagonal elements $A_{ii}$ equal the number of bonds emanating from the *i*th monomer, while the off-diagonal elements $A_{ij}$ are either −1 if monomers *i* and *j* are directly connected by a bond or 0 otherwise. The dynamics of the whole network is described by the set of *N* linearly independent Langevin equations, which for a bead *i* have the following form [15,53,54,58]:

(2) $$\zeta \frac{\partial R_{i}\left(t\right)}{\partial t}+K\sum_{j=1}^{N}AR_{j}\left(t\right)=f_{i}\left(t\right)+F_{i}\left(t\right)$$

where the friction constant of the beads, ζ=6πρa, is formulated in terms of an effective radius *a* and the viscosity of the solvent ρ, and A is (as before) the connectivity matrix of the GGS. In Equation (2), $f_{i}$ represents the stochastic forces that act on the *i*th bead, and as a result of the fluctuation–dissipation theorem, these forces are connected with the dissipative force (or friction). This random forces arise as a result of the incessant collisions of the solvent molecules with the bead, and this is taken to be a Gaussian process with zero mean value $\langle f_{i}\left(t\right)\rangle =0$ and $\langle f_{i\alpha }\left(t\right)f_{j\beta }\left(t^{\prime }\right)\rangle =2k_{B}T\zeta \delta_{ij}\delta_{\alpha \beta }\delta \left(t-t^{\prime }\right)$ (with α and β denoting the x,y, and *z* directions and i,j corresponding to the monomers’ numbers). Any external force acting on the bead *i* is represented by $F_{i}$.

The Rouse model assumes that the relaxation time associated with each mode has the same temperature dependence. The relaxation time of each mode is the product between temperature-independent factors and the monomeric relaxation time (Kuhn monomer relaxation time). The monomeric relaxation time is defined as $\tau_{0}=\zeta /K$. The spring constant *K* has an entropic nature, and it is defined as $K=\frac{3k_{B}T}{l^{2}}$, where *T* is the temperature and $k_{B}$ is the Boltzmann constant. Thus, $\tau_{0}=\frac{\zeta l^{2}}{3k_{B}T}$. The monomeric time $\tau_{0}$ is inversely proportional to the temperature. Therefore, the change of the temperature leads to a shift along the *x* axis, if it has a logarithmic scale.

Classical rheological experiments focus on the investigation of the mechanical relaxation of polymers. We recall that the mechanical relaxation is described by the complex dynamic modulus $G^{*}\left(\omega \right)$ with its real part $G^{\prime }\left(\omega \right)$ (storage modulus) and imaginary part $G^{\prime \prime }\left(\omega \right)$ (loss modulus) [15,54]. In the GGS model, for very dilute solutions and for positive values of the frequency, the mechanical moduli are given by the following (see also Equations (4.159) and (4.160) of [54]):

(3) $$G^{\prime }\left(\omega \right)=\nu k_{B}T\frac{1}{N}\sum_{i=2}^{N}\frac{\omega^{2}}{\omega^{2}+{\left(2\sigma \Lambda_{i}\right)}^{2}}$$

and

(4) $$G^{\prime \prime }\left(\omega \right)=\nu k_{B}T\frac{1}{N}\sum_{i=2}^{N}\frac{2\sigma \omega \Lambda_{i}}{\omega^{2}+{\left(2\sigma \Lambda_{i}\right)}^{2}}$$

where ν represents the number of polymer segments (beads) per unit volume, *T* denotes the temperature, $k_{B}$ is the Boltzmann constant, σ=K/ζ is the inverse of the monomeric relaxation time, and $\Lambda_{i}$ are the eigenvalues of the connectivity matrix A. In these equations, the sum runs over all the eigenvalues, except the vanishing eigenvalue ($\Lambda_{1}=0$), which corresponds to the whole translation of the system. Additionally, for concentrated solutions (when the entanglement effects are negligible), Equations (3) and (4) are still valid, the only change being in the value of $\nu k_{B}T$ [74]. The factor 2 in the relaxation times $\tau_{i}=1/2\sigma \Lambda_{i}$ arises from the second moment of the displacements involved in computing the stress [54]. For these moduli, we are mostly interested in the slopes; thus we compute the results in terms of the reduced storage and loss moduli by setting $\nu k_{B}T/N=1$ and σ=1 in Equations (3) and (4).

As we have mentioned in the introduction, the energy transfer rate by the dipole–dipole coupling mechanism has a $1/r^{6}$ dependence on the donor–acceptor separation distance *r*. This suggests clearly that the excitation is transferred most likely between nearest-neighbor chromophores. Given the construction of the connectivity matrix, which accounts only for connections between nearest neighbors, it shows exactly for each chromophore that holds the excitation the most probable chromophores to which the excitation can be transferred. Moreover, as we show in the following, the eigenvalue spectrum of the connectivity matrix is essential in the calculation of the returning probability. Therefore, any change in the geometry of the structure will be reflected in the connectivity matrix and thus in its eigenvalue spectrum. Such a change in the eigenvalue spectrum may influence the dynamical behavior of the investigated quantity.

Here, we assume that the energy is resonantly exchanged only between nearest neighbors. Moreover, it was shown in [75] for the case of chromophores whose dipolar moments are oriented randomly but fixed in time (frozen) that the transfer among them depolarizes very efficiently both in 3D and also in 2D. Thus, measuring the polarization of the spontaneously emitted fluorescence permits us to determine the probability that the donor excited at time $t_{0}$ is still or is again excited at a later time *t*. Under these assumptions, the dipolar quasi-resonant energy transfer among the chromophores obeys the master equation:

(5) $$\frac{dP_{i}\left(t\right)}{dt}={\sum_{j=1}^{N}}^{\prime }T_{ij}P_{j}\left(t\right)-\left({\sum_{j=1}^{N}}^{\prime }T_{ji}\right)P_{i}\left(t\right)$$

where $P_{i}\left(t\right)$ is the probability for bead *i* to be excited at time *t*, $T_{ij}$ denotes the transfer rate from bead *j* to bead *i*, and the prime sign indicates that the case j=i is excluded from the sum. In Equation (5), the radiative decay is not included, as it leads, in fact, only to the multiplication of all the $P_{i}\left(t\right)$ by $exp(-t/\tau_{d})$, where $1/\tau_{d}$ is the decay rate. Taking now all microscopic transfer rates to nearest neighbors to be equal, for example, *k*, Equation (5) reads

(6) $$\frac{dP_{i}\left(t\right)}{dt}=-k{\sum_{j=1}^{N}}^{\prime }A_{ij}P_{j}\left(t\right)-\left(kA_{ii}\right)P_{i}\left(t\right)$$

with the matrix A being defined as above. In order to solve Equation (6), one has to diagonalize the connectivity matrix A, and, thus, the solution for a given $P_{i}\left(t\right)$ depends both on the eigenvalues and on the eigenvectors of A. However, by averaging over all beads, a procedure that is fully justified when the dipolar orientations are independent of the position of beads in the system, the average probability of finding the excitation at time *t* on the originally excited chromophore depends only on the eigenvalues of the matrix A and is given by [41]:

(7) $$\langle P\left(t\right)\rangle =\frac{1}{N}\sum_{j=1}^{N}\exp\left(-k\Lambda_{j}t\right)$$

It is noteworthy that the average probability 〈P(t)〉 is similar to the quantity $G^{s}\left(t\right)$, the “survival probability” or the ensemble-averaged probability that an excitation resides on the originally excited molecule at time *t* [76,77,78]. It contains contributions from excitations that never leave the originally excited chromophore and from excitations that return to the initially excited chromophore at the later times after one or more transfer events. The average probability does not contain loss of excitation due to lifetime (fluorescence) events. The usefulness of the average probability lies in its connection with the experiment. It can be obtained experimentally from time-resolved fluorescence depolarization measurements by [79,80,81].

In other physicochemical contexts, several quantities can be calculated only by making use of the eigenvalues of the connectivity matrix. In polymer science, forms akin to Equations (3), (4) and (7) describe the stretching of the monomer under a constant external force [43,45,47], and dielectric and magnetic relaxation phenomena [42,62,63]. Such forms turn out to be extremely helpful in relating the dynamical properties to the underlying geometry of the system. This has been possible because the density of eigenvalues ρ(λ), also called the spectral density, reflects the connectivity of the GGS; for isotropic and locally homogeneous fractals, ρ(λ) obeys the power law $\rho \left(\lambda \right)∼\lambda^{(d_{s}-2)/2}$ [82], from which one observes that the connectivity of the fractal object is characterized by one parameter, the spectral dimension $d_{s}$, a quantity directly related to the eigenvalue spectrum.

## 3. The Multilayer Network and the Eigenvalue Spectrum

**Figure 1:**
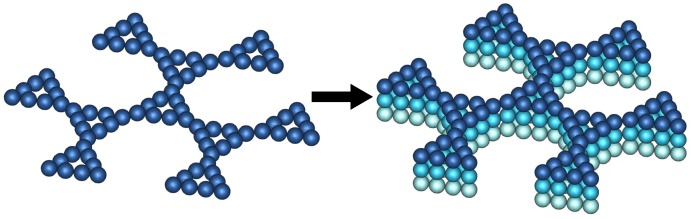
**Left-hand side**: the multihierarchical layer at generation $\left(g_{d}=2,g_{s}=2\right)$. **Right-hand side**: the multilayer network at generation $\left(g_{d}=2,g_{s}=2,n_{l}=3\right)$.

In this section, we show the procedure for building the multilayer network and then present the iterative method, whereby the whole eigenvalue spectrum of the connectivity matrix can be obtained. The construction of the multilayer network is achieved in two steps. The first step consists of the construction of one multihierarchical layer, which represents the basic structure. The general proceduce for building the multihierarchical layer at any desired generation $\left(g_{d},g_{s}\right)$ is based on the replication of the dual Sierpinski gasket (at generation $g_{s}$) in the form of a regular dendrimer (at generation $g_{d}$). Specifically, in the first phase, one replaces each bead of the dendrimer (at generation $g_{d}$) with an arrangement of beads in the shape of a dual Sierpinski gasket (at generation $g_{s}$), and in the second phase, one has to connect with bonds all these identical arrangements in the regular dendrimer form. The left-hand side of Figure 1 displays the basic multihierarchical layer at generation $g_{d}=2$ and $g_{s}=2$. As a result of geometrical restrictions, we strictly use for the construction of the multihierarchical layer dendrimers with f=3 (*f* being the functionality) and dual Sierpinski gaskets with d=2 (*d* being the Euclidean dimension). In the second step, $n_{l}$ identical copies of the basic multihierarchical layer are interconnected. Specifically, each bead of an internal layer, for example, layer *j*, is connected with its corresponding beads from the nearest-neighbor layers, j−1 and j+1. Each bead of the peripheral layer is connected with its corresponding bead from the nearest-neighbor layer. In this way, each bead from an internal layer obtains two additional connections, whereas each bead belonging to a peripheral layer obtains only one additional connection. The right-hand side of Figure 1 displays the multilayer network consisting of three layers, each at generation $g_{d}=2$ and $g_{s}=2$. Throughout this paper, we use only networks built from identical layers, and the generation of a network is described by the parameter set $\left(g_{d},g_{s},n_{l}\right)$, where $g_{d}$ represents the generation of the dendrimer component, $g_{s}$ denotes the generation of the dual Sierpinski component, and $n_{l}$ is the number of layers.

On the basis of the model assumptions and existing experimental data, we provide a rough estimate of our network sizes. The thickness of the layer is due to a bead and a spring and is given by the length of the Kuhn segment. It is in the order of few nanometers. Now, in order to estimate the size of the layer, we use the data reported by Shang et al. [38] for the Sierpinski gasket. They reported for the length of the side of the Sierpinski gasket at generation 3 a value of 33 nm. Using this value, the diameter of the multihierarchical layer at generation $\left(g_{d}=2,g_{s}=3\right)$ is roughly 5 times the length of the side of the equilateral triangle, 165 nm. The estimated diameter of the layer at generation $\left(g_{d}=2,g_{s}=3\right)$ is larger than that of the polyamidoamine (PAMAM) dendrimer of generation 10, which is about 135 nm. Given the fact that the model does not account for excluded volume interactions, such an estimate should be taken to be questionable.

The total number of beads of a dendrimer at generation $g_{d}$ is $N_{d}=3\times 2^{g_{d}}-2$, and the number of beads of a dual Sierpinski fractal at generation $g_{s}$ is $N_{s}=3^{g_{s}}$; hence, the total number of beads of the multilayer network consisting of $n_{l}$ layers is $N=n_{l}\cdot N_{L}$, where $N_{L}=3^{g_{s}}\cdot \left(3\times 2^{g_{d}}-2\right)$ is the total number of beads of a single layer. We recall that the fractal and the spectral dimensions for the dual Sierpinski gasket with d=2 are given by

(8) $$d_{f}=\frac{\ln3}{\ln2}=1.58496\ldots$$

and

(9) $$d_{s}=\frac{2\;\ln3}{\ln5}=1.36521\ldots$$

We now turn our attention to the determination of the eigenvalues of the multilayer network, eigenvalues that will be used to evaluate the dynamical quantities according to Equations (3), (4) and (7). Simply following the construction procedure of the multilayer network, one observes that the connectivity matrix A is a block matrix of size $\left(n_{l}\cdot N_{L}\right)\times \left(n_{l}\cdot N_{L}\right)$. We decompose the matrix A into blocks of square submatrices $A_{\mathrm{ij}}$, each of size $N_{L}\times N_{L}$. Under this observation, the connectivity matrix A has the off-diagonal blocks equal, $A_{\mathrm{ij}}=-I$, if layers *i* and *j* are connected, and $A_{\mathrm{ij}}=O$ otherwise. The diagonal blocks are given by $A_{\mathrm{ii}}=A_{\mathrm{layer}}+2I$ if *i* is an inner layer, $1<i<n_{l}$, and by $A_{\mathrm{ii}}=A_{\mathrm{layer}}+I$ if *i* is a peripheral layer, namely, i=1 or $i=n_{l}$, where $A_{\mathrm{layer}}$ represents the connectivity matrix of a single layer. We exemplify the procedure for a network built from three layers (as displayed by the right-hand side of Figure 1), for which the connectivity matrix is a 3×3 block matrix, and by using the Kronecker product, it can be simplified to

(10) $$A=\left(\begin{array}{ccc} A_{\mathrm{layer}}+I & -I & O\\ -I & A_{\mathrm{layer}}+2I & -I\\ O & -I & A_{\mathrm{layer}}+I \end{array}\right)=A_{\mathrm{linear}}⊗I+I⊗A_{\mathrm{layer}}$$

where $A_{\mathrm{linear}}$ represents the connectivity matrix of a linear chain. We denote the whole eigenvalue spectrum of the multilayer network as $\Delta_{ML}=\left(\Delta_{0},\Delta_{1},\ldots ,\Delta_{j},\ldots ,\Delta_{n_{l}-1}\right)$, where $\Delta_{0}$ contains the first $N_{L}$ eigenvalues (i.e., one layer’s size), $\Delta_{1}$ contains the next $N_{L}$ eigenvalues, and so on, until the last layer, $n_{l}-1$. Following the arguments of [1] for the modes of $A_{\mathrm{linear}}$, the eigenvalues of the multilayer network can be written as

(11) $$\Lambda_{j}=2-2\cos\left(\frac{j\pi }{n\_l}\right)+\lambda_{i}$$

with $j=0,1,\ldots ,n_{l}-1$ numbering the layers. The first two terms of Equation (11) represent the eigenvalues of a linear chain [1], while $\lambda_{i}$ are the eigenvalues of the connectivity matrix $A_{\mathrm{layer}}$ of a single multihierarchical layer with $i=1,\ldots ,N_{L}$. The above approach has considerably simplified the problem and leads to the determination of the eigenvalues of a linear chain and of a single layer. For the first, the procedure is straightforward, as all terms are known; the issue that still remains is obtaining the whole eigenvalue spectrum of one layer. Fortunately, the multihierarchical layer admits an iterative method for the determination of the whole eigenvalue spectrum of its connectivity matrix. We have performed our calculations following the framework of [10,11,15,16,64]. The determination of the eigenvalue spectrum of a single multihierarchical layer is achieved in two distinct stages. Given the multihierarchical layer at any generation $\left(g_{d}>1,g_{s}>1\right)$, the first stage consists of the determination of the eigenvalue spectrum of its dendrimer constituent component. The determination of the eigenvalue spectrum of a dendrimer is based on the fact that the normal modes can be categorized into two general groups: normal modes involving bead motions with a mobile central monomer (which is also called core), and normal modes involving motions with an immobile core. The procedure is summarized in Appendix A.

In the second stage, we determine the whole eigenvalue spectrum of the multihierarchical layer. Here, we follow the analysis of [64]. In this regard, the multihierarchical layer at generation $\left(g_{d},g_{s}\right)$ has to be systematically reduced up to a regular dendrimer of generation $g_{d}$. Such a transformation is achieved by a systematic decimation of the dual Sierpinski gasket component. The decimation procedure is based on the property that the dual Sierpinski gasket, being a self-similar structure, rescales under real-space renormalization transformations. Fundamental in this respect is the following polynomial [64]:

(12) $$P\left(\lambda \right)=-\lambda^{2}+5\lambda$$

One finds, namely, that a part of the eigenvalues of the layer at generation $\left(g_{d},g_{s}\right)$ is obtained from those at generation $\left(g_{d},g_{s-1}\right)$ through the iterative relation

(13) $$P\left(\lambda_{i}^{\left(g_{d},g_{s}\right)}\right)=\lambda_{i}^{\left(g_{d},g_{s}-1\right)}$$

By solving Equation (13), we find the relation between the eigenvalues belonging to consecutive generations of the layer

(14) $$\lambda_{\pm }^{\left(g_{d},g_{s}\right)}=\frac{5\pm \sqrt{25-4\cdot \lambda^{\left(g_{d},g_{s-1}\right)}}}{2}$$

Equation (14) shows that each previous eigenvalue $\lambda_{i}^{\left(g_{d},g_{s}-1\right)}\neq 0$ gives rise to two new eigenvalues at generation $\left(g_{d},g_{s}\right)$. The general procedure for obtaining the whole eigenvalue spectrum of the connectivity matrix of a single layer at any generation $\left(g_{d},g_{s}\right)$ can be summarized as follows: a part of the eigenvalue spectrum is calculated from the eigenvalues of generation ($g_{d}$, $g_{s-1}$) by using Equation (14); these eigenvalues are complemented by the nondegenerate vanishing eigenvalue $\lambda_{1}=0$, $\Gamma_{3}^{g_{d},g_{s}}$ degenerate eigenvalues equal to 3 each, and $\Gamma_{5}^{g_{d},g_{s}}$ degenerate eigenvalues equal to 5 each, where the degeneracies $\Gamma_{3}^{g_{d},g_{s}}$ and $\Gamma_{5}^{g_{d},g_{s}}$ are given by

(15) $$\Gamma_{3}^{g_{d},g_{s}}=2^{g_{d-1}}\cdot \left(3^{g_{s}}+3\right)-3^{g_{s-1}}$$

and

(16) $$\Gamma_{5}^{g_{d},g\_s}=2^{g_{d}-1}\cdot \left(3^{g_{s}}-3\right)-3^{g_{s}-1}+1$$

It is noteworthy that the above procedure makes it clear that the new eigenvalues, determined through Equation (14), keep the degeneracy of their predecessors. Additionally, we mention that the eigenvalues of the dendrimer are used in their form only for determining a part of the spectrum at generation $\left(g_{d},g_{s}=1\right)$; for further generations $\left(g_{d},g_{s}\geq 2\right)$ those obtained from them are used. Moreover, the Appendix B presents a checking of the iterative procedure with respect to the total number of eigenvalues.

In the determination of the eigenvalue spectrum, special attention should be paid to the type of the eigenvalues. The eigenvalue spectrum of the layer consists of persistent and nonpersistent eigenvalues, which form two classes. The term persistent eigenvalue indicates an eigenvalue that appears at a generation and continues to appear in all subsequent generations. Instead, a nonpersistent eigenvalue is an eigenvalue that appears at one generation and is not present in all other subsequent generations. The class of persistent eigenvalues is formed by the eigenvalues obtained from the dual Sierpinski gasket component of the layer. These are the main eigenvalues 3 and 5 and all those determined from them in the subsequent generations of the layer, as well as the nondegenerate vanishing eigenvalue $\lambda_{1}=0$. The class of nonpersistent eigenvalues consists of the eigenvalues of the connectivity matrix of the dendrimer and all those that have resulted from them, on the basis of Equation (14), in the subsequent generations of the layer.

The smallest eigenvalue of the connectivity matrix of a single multihierarchical layer at generation $\left(g_{d},g_{s}\right)$ may be approximated through the following analytical expression [64]:

(17) $$\lambda_{\min}^{\left(g_{d},g_{s}\right)}\approx 5^{-g_{s}}\cdot 2^{-(g_{d}+1)}$$

and for a linear chain consisting of $N_{c}$ monomers, it is approximated by

(18) $$\lambda_{\min}^{\left(c\right)}\approx \frac{\pi^{2}}{N_{c}^{2}}$$

Consequently, the maximal Rouse relaxation time of a single layer and of a linear chain can be estimated through

(19) $$\tau_{RL}\approx \tau_{0}\cdot 5^{g_{s}}\cdot 2^{g_{d}+1}$$

and

(20) $$\tau_{RC}\approx \tau_{0}\frac{N_{c}^{2}}{\pi^{2}}$$

where $\tau_{0}=\zeta /K$ is the monomeric relaxation time.

Before presenting our results, we exemplify the usefulness of the spectrum analysis for investigating different polymer systems. For fractal polymers, the structural and dynamical quantities, calculated on the basis of the eigenvalues, are shown by their time/frequency domain power law behavior [15,42,43]. The power law exponent depends solely on the spectral dimension of the fractal. Instead, for dendritic structures, one expects a logarithmic behavior in the intermediate time/frequency domain of the dynamical quantities [11,15,16]. A very interesting situation was reported in [48] for the structural characterization of randomly hyperbranched polymers of type $AB_{2}$. Their radius of gyration, computed on the basis of the eigenvalues, showed two different behaviors depending on the reaction pathway. The radius of gyration of the structures obtained by adding reactive monomers sequentially showed a logarithmic behavior as imperfect dendrimers, while for the structures obtained by the step reaction allowing for cluster–cluster aggregation, it showed power law behavior as random fractals.

## 4. Relaxation Patterns

### Results

**Figure 2:**
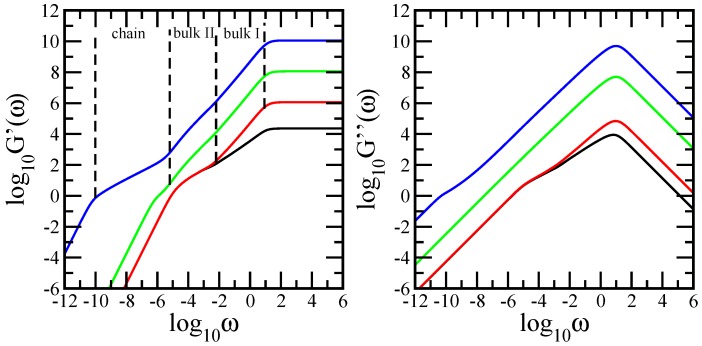
The storage modulus (**right-hand side**) and the loss modulus (**left-hand side**) for the multilayer network at generations ($g_{d}=5$, $g_{s}=5$, $n_{l}=1$), ($g_{d}=5$, $g_{s}=5$, $n_{l}=50$), ($g_{d}=5$, $g_{s}=5$, $n_{l}=5000$), and ($g_{d}=5$, $g_{s}=5$, $n_{l}$ = 500,000).

With the eigenvalues determined through iterative means, we are now able to compute the different quantities introduced in Section 2. Most measurements on polymers are monitored in the frequency domain; furthermore, they involve macroscopic changes. Given the relative ease by which mechanical relaxation measurements can nowadays be performed, we focus on the relaxation moduli $G^{\prime }\left(\omega \right)$ and $G^{\prime \prime }\left(\omega \right)$, given by Equations (3) and (4), in which we have set $\nu k_{B}T/N=1$ and σ=1. The left-hand-side panel of Figure 2 shows the results obtained for the storage modulus, while the right-hand-side panel displays the results obtained for the loss modulus. We mention that the mechanical relaxation quantities are presented in dimensionless units. In both panels of Figure 2, we use multilayer networks at generation $\left(g_{d}=5,g_{s}=5\right)$ and consisting of 50 layers (results denoted with red solid line), 5000 layers (green solid line), and 500,000 layers (blue solid line). Additionally, in both panels, the black solid line denotes the results achieved for a single-layer network. Consequently, the total number of monomers in the network varied from *N* = 22,842 to *N* = 11,421,000,000. In Figure 2, it is straightforward to identify those regions that are not associated with the microscopic structure of the system, which can be observed in the very high and very low frequency parts of the spectrum. For very high ω, we find $G^{\prime }\left(\omega \right)∼\omega^{0}$ and $G^{\prime \prime }\left(\omega \right)∼\omega^{-1}$, which indicates a single-bead mechanical response, whereas low frequencies $G^{\prime }\left(\omega \right)∼\omega^{2}$ and $G^{\prime \prime }\left(\omega \right)∼\omega$ represent the mechanical response of the entire multilayer network. However, neither the very low nor the very high frequency domains are typical for the multilayer polymer network under investigation; the microscopic characteristics of the network are revealed in the intermediate frequency domain. Remarkably, the intermediate frequency domain reveals a clear transition from single-layer behavior to chain-like behavior as the number of layers increased. For a single multihierarchical layer, the intermediate frequency domain of the relaxation moduli split into two regions. The region situated at lower intermediate frequencies appears as a concave curvature, which in the double logarithmic scales of the figure, indicates no scaling; this is a typical relaxation behavior for dendritic-like structures (modified dendrimers, dendritic wedges or regular dendrimers). Given the fact that we had a regular dendrimer, we infer that this region corresponds to its relaxation dynamics. The curves in the intermediate region of higher frequencies appear as straight lines showing power law behaviors: $G^{\prime }\left(\omega \right)∼\omega^{\alpha^{\prime }}$ and $G^{\prime \prime }\left(\omega \right)∼\omega^{\alpha^{\prime \prime }}$. Linear fits in the scaling region led to $\alpha^{\prime }=0.694$ and $\alpha^{\prime \prime }=0.641$. From the comparison of the obtained slopes with the theoretical value $d_{s}/2=0.68261$, calculated from Equation (9), this results precisely in that the region corresponds to the mechanical relaxation of the dual Sierpinski gasket component of the multihierarchical layer. The obtained global dynamic behavior is in line with the original scaling behavior of the dual Sierpinski gasket and with the original non-scaling behavior of the dendrimer. Thus, the multihierarchical layer retained the original behaviors of its components. For the 50-layer network, the intermediate frequency domain consists of three regions. The region located at higher intermediate frequencies appears as a straight line, which denotes scaling behavior. The obtained values of the slopes are $\alpha^{\prime }=1.189$ and $\alpha^{\prime \prime }=0.89$. The first value is very close to the sum of half the spectral dimension of the dual Sierpinski gasket and half the spectral dimension of a linear chain, $d_{s}/2+\tilde{d}/2=1.18261$, where $\tilde{d}=1$ represents the spectral dimension of a linear chain and $d_{s}$ is given by Equation (9). This suggests clearly a bulk-like relaxation behavior, a mixture between the dual Sierpinski gasket and linear chain. In the figure, the region is indicated with bulk I. A very short region at the end of the scaling region indicates the beginning of a second bulk-like behavior. This means that the dual Sierpinski gasket component was relaxed but the linear chain was not entirely. Therefore, the second bulk-like behavior was a mixture between the dendrimer component and the linear chain. The second bulk is indicated with bulk II. The region situated at the lower intermediate frequency domain corresponds to the relaxation of only the dendrimer component. This is justified from the perfect overlapping with the behavior of the dendrimer component of a single layer. Going further to the multilayer network consisting of 5000 layers, one observes that the intermediate frequency domain decomposes into three regions. The region situated at higher intermediate frequencies corresponds to the first bulk-like relaxation. The second bulk involving the cooperative relaxation of the dendrimer and chain extends over the medium intermediate frequencies. Then, at lower intermediate frequencies, one observes a short region corresponding to the relaxation of the linear chain. This region is due to the fact that the dendrimer component was entirely relaxed but the chain needed more time to fully relax. The entire transition from the pure layer to the linear chain is best observed in the intermediate frequency domain of the multilayer network consisting of 500,000 layers. In the region of the first bulk, the slopes of the curves are $\alpha^{\prime }=1.184$ and $\alpha^{\prime \prime }=0.97$. Besides the first and the second bulk, which are well rendered, the region located at lower intermediate frequencies extends over 4 orders of magnitude and appears as a straight line with slope 0.5. This indicates a clear linear-chain behavior. We note that the accuracy obtained for $G^{\prime }\left(\omega \right)$ is better than that obtained for $G^{\prime \prime }\left(\omega \right)$. This is due to the fact that $G^{\prime \prime }\left(\omega \right)$ is, in general, a less-sensitive measure of the relaxation than $G^{\prime }\left(\omega \right)$ [42,45]. Moreover, the slope of $G^{\prime \prime }\left(\omega \right)$ bounds the expression $d_{s}/2+\tilde{d}/2=1.18261$ from below, whereas the slope of $G^{\prime }\left(\omega \right)$ approaches the expression from above.

**Figure 3:**
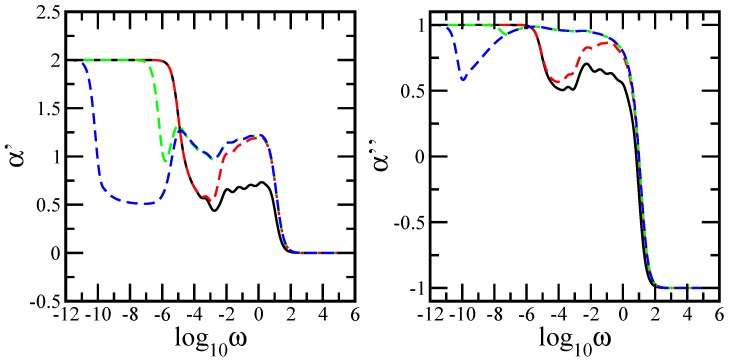
**Left-hand-side panel**: Local slopes $\alpha^{\prime }$ of the curves of Figure 2. **Right-hand-side panel**: Local slopes $\alpha^{\prime \prime }$ of the curves of Figure 2.

In order to render the transition more quantitatively, we have plotted in Figure 3 the local slopes of the curves of Figure 2. The left-hand-side panel of Figure 3 displays the quantity $\alpha^{\prime }=d\left(\log_{10}G^{\prime }\left(\omega \right)\right)/d\left(\log_{10}\omega \right)$, and the right-hand-side panel displays the quantity $\alpha^{\prime \prime }=d\left(\log_{10}G^{\prime \prime }\left(\omega \right)\right)/d\left(\log_{10}\omega \right)$. Their analytical expressions are given by

(21) $$\alpha^{\prime }=\frac{8\sigma^{2}\sum_{i=2}^{N}\frac{\Lambda_{i}^{2}}{{\left(\omega^{2}+4\sigma^{2}\Lambda_{i}^{2}\right)}^{2}}}{\sum_{i=2}^{N}\frac{1}{\omega^{2}+4\sigma^{2}\Lambda_{i}^{2}}}$$

and

(22) $$\alpha^{\prime \prime }=\frac{\sum_{i=2}^{N}\frac{4\sigma^{2}\Lambda_{i}^{3}-\omega^{2}\Lambda_{i}}{{\left(\omega^{2}+4\sigma^{2}\Lambda_{i}^{2}\right)}^{2}}}{\sum_{i=2}^{N}\frac{\Lambda_{i}}{\omega^{2}+4\sigma^{2}\Lambda_{i}^{2}}}$$

In both panels of Figure 3, the *x*-axis is logarithmic to basis 10, and the *y*-axis is linear. The limiting connectivity behaviors are well rendered, namely, $\alpha^{\prime }=0$ and $\alpha^{\prime \prime }=-1$ for very high ω and $\alpha^{\prime }=2$ and $\alpha^{\prime \prime }=1$ for very small ω. Our main focus is the intermediate frequency domain. For the single-layer network (black solid line), in both panels of the figure, the in-between frequency domain consists of two regions: one region with a decreasing slope corresponding to the dendrimer component, followed at larger intermediate frequencies by a plateau region corresponding to the dual Sierpinski gasket component. The average values of each plateau are $\alpha^{\prime }=0.694$ and $\alpha^{\prime \prime }=0.641$. Increasing the number of layers to 50 (red dashed line), the first important observation is the formation of the first bulk (dual Sierpinski gasket and the linear chain). The linear chain contribution increased the average plateau values to $\alpha^{\prime }=1.189$ and $\alpha^{\prime \prime }=0.89$. At the end of the plateau, towards lower frequencies, one observes a slight increase in the local minimum compared to the single-layer structure. This indicates that the second bulk began to form. At much lower intermediate frequencies, the pure dendrimer behavior is evident; here, the curve overlaps the region that corresponds to the dendrimer behavior of a single-layer network. The formation of the second bulk is emphasized by the lower intermediate frequency region of the multilayer structure consisting of 5000 layers (green dashed line). Moreover, at the end of the second bulk, toward much lower frequencies, one notes the appearance of a minimum. This is due to the fact that the linear chain behavior began to dominate at longer relaxation times. Increasing the number of layers further to 500,000 (blue dashed line), the minimum became stabilized at the value 0.5, and the frequency interval became broader. This is well rendered in the figure by the plateau value 0.5, which extends over several orders of magnitude and indicates a clear chain-like behavior. Again, the elastic part was more sensitive than the dissipative part. Furthermore, oscillations due to the local structure and multihierarchical construction are evident in the figure. This is a sign of the underlying Cantor-set structure of the spectrum; the sums in Equations (21) and (22) could no longer smooth out the structure. Such waviness has been observed in [42] and [43].

**Figure 4:**
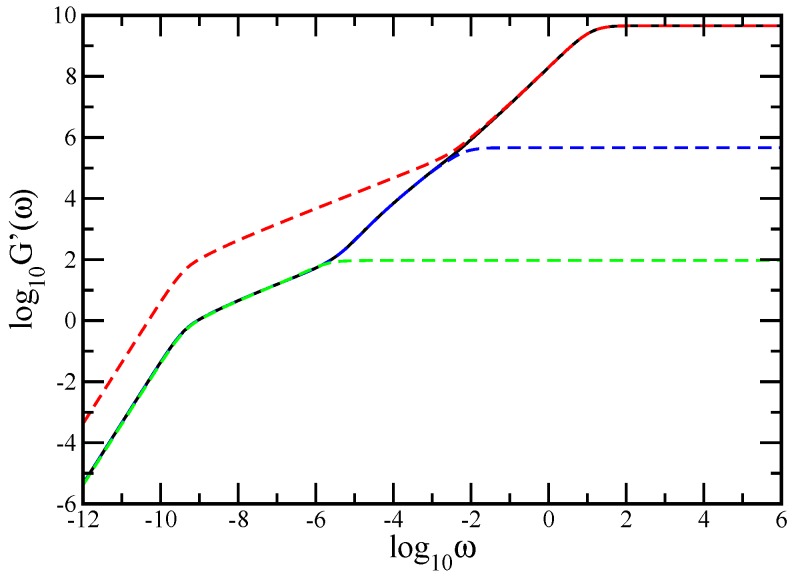
The storage modulus for the multilayer network at generation ($g_{d}=5,g_{s}=5,n_{l}$ = 200,000), the multilayer network based on dual Sierpinski gasket ($g_{s}=5$, $n_{l}$ = 200,000), the multilayer network based on dendrimer ($g_{d}=5,n_{l}$ = 200,000), and a linear chain of 200,000 monomers.

Figure 4 shows in an explicit manner the reflection of the composition of each bulk, as well as the linear chain of macromonomers, in the dynamical behavior of the storage modulus. We display comparatively the results obtained for the original multilayer network at ($g_{d}=5,g_{s}=5$, $n_{l}$ = 200,000), shown by the black solid line; a multilayer network consisting of 200,000 layers (each a dual Sierpinski gasket at $g_{s}=5$), shown by the red dashed line; a multilayer network of 200,000 dendrimers, each at $g_{d}=5$, shown by the blue dashed line; and a linear chain consisting of 200,000 beads, shown by the green dashed line. We note that, for matching, the number density of the dual Sierpinski gaskets is the same as in the original multilayer network; the friction coefficient of each bead in the dendrimer is equal to the total friction coefficient of the pure dual Sierpinski gasket $\left(g_{s}=5\right)$ in the original multilayer network, and the friction coefficient of each bead of the chain equals the total friction coefficient of the beads of a single layer of the original multilayer network. The results obtained for the multilayer networks based on the pure dual Sierpinski gasket and on the pure dendrimer perfectly match the corresponding results of the original multilayer network. This explicitly highlights the bulks and strengthens the idea about their compositional fractal chain and, respectively, dendrimer chain. Furthermore, for the multilayer network consisting of an extremely large number of layers, the chain-like behavior observed at very long relaxation times is explicitly confirmed by the overlapping with the relaxation behavior of a pure linear chain.

The theoretical findings are well supported by mechanical relaxation experiments, both with respect to scaling and to the splitting of the intermediate frequency domain into regions with different maximal relaxation times. The experimental results reported by the authors of [83] for the end-functionalized polymer melts and by the authors of [70] for the coexistence of long and short polymer-like micelles in lecithin organogel are in very good agreement with our theoretical results for both the scaling and decomposition of the intermediate frequency domain. The authors reported a slope of around 0.5 in the storage and loss moduli at the lower part of the intermediate frequency domain as well as two relaxation times in this domain. Similar behaviors in the intermediate frequency domain have been reported for complex supramolecular dendritic polymer networks in melt state [84,85], associative polymer networks [86,87], styrene–isoprene diblock copolymer micelles [88], multifunctional polyhedral oligomeric silsesquioxane (POSS)/poly(propylene oxide) (PPO) nanocomposites [89], and isotropic elastomers [90].

**Figure 5:**
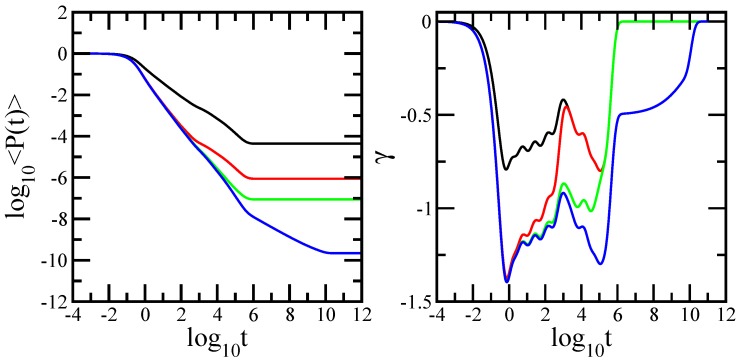
**Left-hand-side panel**: average probability for the multilayer network at generations ($g_{d}=5,g_{s}=5,n_{l}=1$), ($g_{d}=5,g_{s}=5,n_{l}=50$), ($g_{d}=5,g_{s}=5,n_{l}=500$), and ($g_{d}=5$, $g_{s}=5$, $n_{l}$ = 200,000). **Right-hand-side panel**: Local slopes γ of the curves of the left-hand-side panel.

Now, we turn our attention to the dynamical behavior of the average probability 〈P(t)〉, given by Equation (7). We have mentioned in the previous section that the energy is resonantly exchanged only between nearest neighbors. Each nearest neighbor of the excited chromophore (bead) has anequal probability to obtain the excitation. Thus, the energy transfer practically performs an unbiased random walk over the multilayer network of chromophores. In the left-hand-side panel of Figure 5, we present the average probability 〈P(t)〉 that an initially excited chromophore at time $t_{0}$ is still or is again excited at time *t*, and we display the results in double-logarithmic scales. In the same manner as in Figure 3, the right-hand-side panel of Figure 5 shows the quantity $\gamma =d\left(\log_{10}\langle P\left(t\right)\rangle \right)/d\left(\log_{10}t\right)$, the derivatives of the curves of the left-hand-side panel. In the right-hand-side panel of Figure 5, the *x*-axis is logarithmic to basis 10, and the *y*-axis is linear. The employed networks are $\left(g_{d}=5,g_{s}=5,n_{l}=1\right)$, the black solid line; $\left(g_{d}=5,g_{s}=5,n_{l}=50\right)$, the red solid line; $\left(g_{d}=5,g_{s}=5,n_{l}=500\right)$, the green solid line; and ($g_{d}=5,g_{s}=5$, $n_{l}$ = 200,000), the blue solid line. Consequently, their sizes vary from *N* = 22,842 to *N* = 4,568,400,000 chromophores. At very short times, the average probability equals 1. This value corresponds to the situation in which the random walker did not perform the first step. At very long times, the average probability shows a plateau; this behavior is due (in the absence of radiative decay) to the equipartition of the energy over all chromophores in the network, so that each chromophore has a probability 1/N of harboring the excitation. Again, we focus on the intermediate time domain. For a single-layer network, the intermediate time domain of 〈P(t)〉 shows two distinct regions. The region located at shorter intermediate times appears as a straight line, which indicates scaling. Formally this means that the probability is well represented by a power law: $\langle P\left(t\right)\rangle ∼t^{\gamma }$. The best approximation to our data leads to γ=−0.668. From the comparison with the theoretical value $\gamma =-d_{s}/2=-0.68261$, this results in that the region corresponds to the dual Sierpinski gasket component of the layer. In the double-logarithmic plot, the average probability in the region of longer intermediate times develops concave curvature, a fact that indicates dendritic-like behavior. Therefore, at shorter intermediate times, it is more likely to find the excitation within the chromophores belonging to the same dual Sierpinski gasket, whereas at longer intermediate times, it is possible to find the excitation on the chromophores of the different dual Sierpinski gaskets. Because the dual Sierpinski gaskets are connected in terms of dendrimer shape, the resulting behavior of the probability emerges clearly. For the multilayer network built from 50 layers, the intermediate time domain of the average probability also splits into two regions. Differently from the case of a single layer, the region situated at lower intermediate times thus becomes larger and appears as a straight line whose slope is −1.13. This situation resembles that observed in Figure 2 in the study of the mechanical relaxation moduli. The value of the slope is close to half of the sum of the spectral dimensions. In this region, 〈P(t)〉 shows bulk-like behavior, a coexistence of the dual Sierpinski gasket and the linear chain. In this time interval, the random walker most likely returned to chromophores of the same dual Sierpinski gasket, but in the same time, as a result of the construction of the network, it also could move up and down as in a linear chain that connects different layers. At the longer intermediate times, the average probability shows dendritic-like behavior. In this time interval, the random walker could explore the whole layer. Practically, it had sufficient time to return, in average, to each chromophore of the layer. In the derivative, the curve overlaps with that corresponding to the single-layer network. For the multilayer network consisting of 500 layers, one observes in the region of large intermediate times, the formation of the second bulk. Statistically, this means that the random walker could explore and return to each chromophore of several interconnected layers, hence indicating the linear-chain contribution to the dynamical behavior. In the derivative, this region is shifted to higher values compared to that obtained for the single layer. Moreover, the intermediate time region displaying the second bulk is larger than its corresponding regions, for which one observes only pure dendritic-like behavior (the previous two cases). For the multilayer network consisting of 200,000 layers, the intermediate time domain of the average probability divides into three regions: the two bulk-like regions, and, in addition, to the just discussed case, which at very large times is a region that appears as a straight line with slope 0.5. This indicates pure chain-like behavior. Such behavior is explained through the fact that the number of layers is much larger than the number of chromophores of a single layer. Evidently, in such a case, the random walker needs less time to return to each chromophore of the layer than to each chromophore of a huge linear chain. Oscillations due to the local structure and multihierarchical construction are also evident.

If the transition dipoles of the chromophores in a solid polymer matrix are randomly oriented and static, the source of fluorescence depolarization in an experiment will be excitation transport. Thus the experimentally measured time dependence of fluorescence depolarization [79,80,81] will be related to the time-dependent probability that the excitation is at the initial site. It is noteworthy that the average probability is useful for processes such as the kinetics of fluorescence of rare earth ions in solids studied by line-narrowing spectroscopy [91] or the study of energy transfer and trapping in dendrimers [92,93]. Additionally, we mention that multilayer network schemes assume a great importance in 3D complex problems occurring in different fields of science: the evaluation of the persistent concentration of a pollutant when contamination occurs [94]; the dynamics of water, nutrients and pollutants in the vadose zone [95,96] in the area of soil physics; or the study of light interception into plant canopies [97] in plant ecophysiology.

## 5. Conclusions

In this paper we have studied the mechanical relaxation and the fluorescence depolarization under the dipolar quasiresonant energy transfer of a multilayer network built by connecting to each other identical multihierarchical layer on the basis of the dual Sierpinski gasket and regular dendrimer. Both dynamical processes have been investigated in the framework of the GGS model. Our goal was to monitor how the addition of more and more layers influences the network’s dynamics, particularly the way in which the underlying topologies of the whole network are highlighted by the dynamical behavior of the mechanical relaxation moduli and by the average returning probability.

We have developed an iterative method for the determination of the whole eigenvalue spectrum of the connectivity matrix of the multilayer network. It relies on three solid properties: the rescaling of the dual Sierpinski gasket under real space decimation, the symmetries of the regular dendrimer, and the decomposition of the connectivity matrix into blocks of square submatrices. On the basis of the eigenvalues obtained in the iterative manner, we were able to analyze the dynamics of the multilayer networks consisting of a huge number of layers, which is impossible to attain through numerical diagonalizations.

The investigated dynamical quantities have shown a transition from layer-like behavior to chain-like behavior as a function of the number of layers. Remarkably, we have highlighted the existence of two bulk-like behaviors. These are time intervals in which the constituent components of the multilayer network have common relaxation. If the first bulk forms at a rather small number of layers, for the appearance of the second, one needs networks built up from a sufficiently large number of layers. Furthermore, in order to attain the chain-like behavior, one has to interconnect a huge number of layers. From here, the necessity for an iterative method becomes crucial.

Decomposition behavior in the intermediate region of the dynamical quantities has been reported for different multihierarchical structures [46,64,98] and for a 3D cubic network cross-linked from linear chains [15]. These structures preserve the individual behaviors of their components. For them, each region of the intermediate domain shows the typical behavior of only one constituent component. What is different from all these mentioned structures and what makes our multilayer network very interesting are that the intermediate domain shows bulk-type behaviors, the coexistence of the constitutive components of the network, and definitely not only the behavior of a single constituent. For large number of layers, our multilayer network does not preserve the individual behaviors of its constituent components. For small number of layers, it preserves these only partially, for the dendritic component and a few unrelaxed parts of the dual Sierpinski gaskets. Moreover, for the case of the 3D cubic network [15], chain-like behavior is obtained, because the network junctions are connected with linear chains consisting of a certain number of monomers. Instead, for the multilayer network, the layers are not connected with linear chains of a certain number of monomers. The chain-like character results from the fact that each monomer of an internal layer is connected with its corresponding monomer from the neareast-neighbor layers and, when the number of layers is very large, also from the fact that each layer plays the role of a macromonomer of a linear chain.

We have compared our general theoretical features found for the multilayer network with experimental results from the literature. The theoretical findings both with respect to scaling and to the splitting of the intermediate frequency domain into regions with different maximal relaxation times are well supported by the experimental results. In addition to the dynamical quantities studied in this article, in the framework of the GGS model, many other quantities can be calculated by the use of the eigenvalue spectrum of the connectivity matrix. Of these, we recall the mean first passage time of a random walk [99,100], the dielectric relaxation functions [42], and the NMR relaxation functions [62]. Furthermore, such multilayer networks are of great use in other domains of science: in the area of soil physics for the evaluation of the persistent concentration of a pollutant when contamination occurs [94]; the dynamics of water, nutrients and pollutants in the vadose zone [95,96]; and in plant ecophysiology, for the study of light interception into plant canopies [97]. This strengthens the interdisciplinary character of the present research.

We address the multilayer networks as possible theoretical models for the relaxation dynamics of different polymer systems as end-functionalized polymer melts, micelle networks, complex supramolecular dendritic polymer networks, and associative polymer networks.

## Abbreviations

The following abbreviations are used in this manuscript:

$g_{s}$	Generation of dual Sierpinski gasket
$g_{d}$	Generation of the dendrimer
$n_{l}$	Number of layers of the multilayer network
GGS	Generalized Gaussian structure

## Appendix A. Determination of the Eigenvalue Spectrum of the Dendrimer

In the following, we summarize the procedure [10,11,15,16] for determining the eigenvalues of a dendrimer of generation $g_{d}$ having the functionality f=3. All the eigenvalues can be divided into two classes: the symmetric eigenmodes and the nonsymmetric modes. In the first class, there are $g_{d}$ nondegenerate eigenmodes, for which all the monomers are moving, with the eigenvalues determined from the equation

(A1) $$\lambda_{k}^{\left(s\right)}=3-2\sqrt{2}\cos\left(\frac{\pi k}{g_{d}+1}\right),\;\mathrm{with}\;k=1,\ldots ,g_{d}$$

Additionally, we have the overall translational motion, which corresponds to the vanishing eigenvalue $\lambda_{g_{d}+1}^{\left(s\right)}=0$.

The second class of eigenvalues is composed by modes that have an immobile core (central bead), and it contains only degenerate eigenvalues. If only the central bead is immobile, the eigenvalues are double-degenerate, and they are determined by solving a system of two trigonometrical equations:

(A2) $$\lambda_{k}^{\left(n\right)}=3-2\sqrt{2}\cos\psi_{k}$$

and

(A3) $$\sin\left(g_{d}+1\right)\psi_{k}=\sqrt{2}\sin g\_d\psi_{k}$$

For $g_{d}<3$, this system of two equations provides $g_{d}$ distinct solutions. However, for $g_{d}\geq 3$, these equations give only $g_{d}-1$ solutions, and the last solution corresponds to a hyperbolic eigenvalue:

(A4) $$\lambda^{\left(n\right)}=3-2\sqrt{2}\cosh\psi$$

where ψ fulfils the following equation:

(A5) $$\sin h\left(g_{d}+1\right)\psi =\sqrt{2}\sinh g_{d}\psi$$

Usually, there are more monomers that remain immobile, aside from the central node, and the eigenvalues are still given by Equation (A2), but $\psi_{k}$ now obeys

(A6) $$\sin\left(g_{d}+1-m\right)\psi_{k}=\sqrt{2}\sin\left(g_{d}-m\right)\psi_{k}$$

where we denote by *m* (with $0<m<g_{d}-1$) the last generation number for which all the monomers are immobile. From the last equation, one obtains $\left(g_{d}-m\right)$ distinct solutions if $\left(g_{d}-m+1\right)>\sqrt{2}\left(g_{d}-m\right)$. Otherwise, one obtains $\left(g_{d}-m-1\right)$ distinct eigenvalues given by Equation (A2) and one additional eigenvalue of hyperbolic form given by Equation (A4), with ψ being given by the following equation:

(A7) $$\sin h\left(g_{d}-m+1\right)\psi =\sqrt{2}\sin h\left(g_{d}-m\right)\psi$$

Summing up, there are $\left(g_{d}-m\right)$ distinct eigenvalues for every *m*, and every eigenvalue is $3\times 2^{m-1}$-fold degenerate. Finally, for the special case of $m=g_{d}-1$, which corresponds to all pairs of peripheral monomers from the same branch moving against each other, we encounter the eigenvalue λ=1 with a degeneracy equal to $3\times 2^{g_{d}-2}$.

## Appendix B. Checking of the Total Number of Eigenvalues

Here, we show that the iterative method provides the whole eigenvalue spectrum of the connectivity matrix of the multilayer networks. For a single layer at any generation $\left(g_{d},g_{s}\right)$, the total number of nonpersistent eigenvalues is

(A8) $$N_{np}^{g_{d},g_{s}}=2^{g_{s}}\cdot \left(3\times 2^{g_{d}}-3\right)$$

The total number of persistent degenerate eigenvalues obtained from eigenvalue 3 (also including eigenvalue 3) is

(A9) $$N_{3}^{g_{d},g_{s}}=\sum_{i=0}^{g_{s}-1}2^{i}\cdot \Gamma_{3}^{g_{d},g_{s}-i}=2^{g_{d}-1}\cdot \left(3^{g_{s}+1}-3\right)+2^{g_{s}}-3^{g_{s}}$$

The total number of persistent degenerate eigenvalues obtained from eigenvalue 5 (also including eigenvalue 5) is

(A10) $$N_{5}^{g_{d},g_{s}}=\sum_{i=0}^{g_{s}-1}2^{i}\cdot \Gamma_{5}^{g_{d},g_{s}-i}=2^{g_{d}-1}\cdot \left(3^{g_{s}+1}+3\right)+2^{g_{s}+1}\cdot \left(1-3\cdot 2^{g_{d}-1}\right)-3^{g_{s}}-1$$

Then, the total number of modes of a layer at generation ($g_{d}$, $g_{s}$) is

(A11) $$N_{L}=1+N_{np}^{g_{d},g_{s}}+N_{3}^{g_{d},g_{s}}+N_{5}^{g_{d},g_{s}}=3^{g_{s}}\cdot \left(3\cdot 2^{g_{d}}-2\right)$$

where we also have taken into account the nondegenerate eigenvalue $\lambda_{1}$=0.

Now, taking into account that the $N_{L}$ eigenvalues are inserted $n_{l}$ times into Equation (11), we obtain the total number of eigenvalues of the multilayer network at $\left(g_{d},g_{s},n_{l}\right)$:

(A12) $$N=n_{l}\cdot N_{L}=n_{l}\cdot 3^{g_{s}}\cdot \left(3\cdot 2^{g_{d}}-2\right)$$
